# Construction and validation of a nomogram prediction model for antiviral efficacy based on clinical characteristics and intestinal microflora distribution in patients with chronic hepatitis B

**DOI:** 10.3389/fmed.2025.1542104

**Published:** 2025-06-12

**Authors:** Hongjie Wu, Mingqiang Yue, Tianbao Wang, Xiaoxia Wei, Yanping Wang, Changyun Si

**Affiliations:** Department of Infectious Diseases, The First Affiliated Hospital of Xinxiang Medical University, Xinxiang, China

**Keywords:** chronic hepatitis B, antiviral treatment, distribution of intestinal flora, clinical characteristics, nomogram prediction model

## Abstract

**Objective:**

To construct and validate a nomogram prediction model based on clinical characteristics and intestinal flora distribution in patients with chronic hepatitis B.

**Methods:**

Patients with chronic hepatitis B were divided into training set (*n* = 175) and verification set (*n* = 75) according to the ratio of 7:3 by complete random method. In the training set, multivariate logistic regression was used to analyze the risk factors for the failure of antiviral therapy and the nomogram prediction model was constructed. The ROC curve and calibration curve were drawn to evaluate the prediction efficiency of the nomogram model and were verified in the verification set.

**Results:**

There was no significant difference in the incidence, clinical characteristics and distribution parameters of intestinal flora between the training set and the verification set (*p* > 0.05). Univariate analysis showed that the training set treatment ineffective group and the effective group had statistical differences in ALT, AST, hepatitis B virus DNA quantification, Shannon-Wiener index, Simpson index, Chao1 index, ACE index, relative abundance of *Sclerotinia sclerotiorum*, relative abundance of Bacteroides immitis, and PCA clustering separation (*p* < 0.05). Multivariate logistic regression analysis identified AST, hepatitis B virus DNA quantification, Shannon-Wiener index, Simpson index, and the relative abundance of Firmicutes and Bacteroides as independent risk factors for antiviral therapy failure (*p* < 0.05). Further, the nomogram prediction model was constructed, and the nomogram model had good calibration and fitting between prediction and reality in the training set and the verification set (ROC curves were shown in the training set and the verification set); AUC of the nomogram model for predicting the antiviral treatment effect was 0.869 and 0.829.

**Conclusion:**

The nomogram model shows good discriminative ability for predicting suboptimal antiviral response, requiring multicenter validation. It should complement, not replace, clinical judgment and virological monitoring, aiding early risk identification and targeted interventions.

## 1 Introduction

Despite advances in antiviral therapy, heterogeneous treatment responses in CHB remain a clinical challenge, necessitating better predictive tools. According to the data of the World Health Organization, there are about 296 million people infected with chronic hepatitis B virus in the world, and about 820,000 people die from hepatitis B-related diseases every year (1). In China, the prevalence of chronic hepatitis B remains high. Although the new infection rate has decreased with the extensive vaccination of hepatitis B vaccine in recent years, the number of patients with chronic hepatitis B cannot be underestimated due to the large infection base (2, 3). The natural course of chronic hepatitis B is long and complex, which can progress to severe complications such as liver cirrhosis, liver failure and hepatocellular carcinoma, and poses a great threat to the life, health and quality of life of patients (4–6). At present, antiviral therapy is the key measure for the treatment of chronic hepatitis B. It can effectively inhibit hepatitis B virus replication, reduce liver inflammation and fibrosis, and reduce the risk of liver cirrhosis and liver cancer (7). However, it has been found in clinical practice that the therapeutic effects of different patients vary significantly even when receiving the same antiviral therapy. Some patients can achieve the ideal therapeutic goals such as virologic response, serological transformation and liver function improvement, while others have poor therapeutic effects with continuous virus replication and continuous progression of liver lesions (8, 9). The heterogeneity of treatment response impacts patient prognosis and complicates clinical decision-making.

In recent years, with the rapid development of microbiological technology, the role of intestinal flora in the pathogenesis and treatment of chronic hepatitis B has gradually attracted extensive attention. A study has shown that patients with chronic hepatitis B have an imbalanced intestinal flora, which is characterized by decreased intestinal microbial diversity, decreased beneficial bacteria, and increased harmful bacteria (10). Intestinal flora imbalance can affect the progression and therapeutic effect of chronic hepatitis B through a variety of ways. For example, impaired intestinal barrier function leads to bacterial translocation and aggravation of liver inflammation. Abnormal changes of metabolites of intestinal flora, such as short-chain fatty acids and endotoxin, can regulate the immune microenvironment of the liver and affect the antiviral immune response. In addition, the intestinal flora may interact with hepatitis B virus to directly or indirectly affect the replication and removal of the virus (11–13). Therefore, the distribution characteristics of intestinal flora is expected to become an important biomarker for predicting the effect of antiviral treatment for chronic hepatitis B.

As a visual statistical tool, nomogram prediction model can integrate the information of multiple prediction factors, convert complex mathematical models into intuitive and understandable graphics, and provide convenient individual prediction tools for clinicians. By constructing the nomogram prediction model based on the clinical characteristics of patients with chronic hepatitis B and the distribution of intestinal flora, various information of the patients can be comprehensively considered, and the antiviral treatment effect can be predicted more accurately, so that clinicians can identify patients with possible poor treatment effect before treatment and formulate personalized treatment plans for the patients, such as adjusting the types and doses of antiviral drugs or combining other treatment means, improving the effectiveness and pertinence of treatment, better performing disease management and prognosis evaluation on the patients, and reducing unnecessary medical resource waste and economic burden of the patients.

Based on the above background, the purpose of this study was to collect clinical data and intestinal flora data of patients with chronic hepatitis B, screen out the key factors related to the effect of antiviral treatment using multi-factor analysis method, and construct nomogram prediction model, and evaluate the prediction efficiency and reliability of the model through strict internal verification and external verification, to provide a scientific basis and practical tool for the accurate treatment of chronic hepatitis B. To ensure the robustness of our prediction model, we conducted comprehensive baseline comparisons between the training and validation sets, including demographic characteristics, clinical parameters, and intestinal microbiota distribution. These comparisons confirmed the homogeneity of the two sets, thereby enhancing the reliability and generalizability of our model.

## 2 Data and methods

### 2.1 Research objects

Two hundred and fifty patients with chronic hepatitis B in our hospital from 2021 to 2024 were selected as the research subjects, and they all informed consent and voluntarily participated in this study, which was approved by the Ethics Committee of our hospital. Patients with chronic hepatitis B were randomly divided into a training set (*n* = 175) and a validation set (*n* = 75) at a 7:3 ratio using complete randomization, and baseline data were collected synchronously.

### 2.2 Inclusion exclusion criteria

#### 2.2.1 Inclusion criteria

Patients who are aged between 18 and 65 years old, both male and female, with hepatitis B surface antigen positivity lasting for more than 6 months and measurable hepatitis B virus DNA quantitation (lower limit of high sensitivity detection is 20 IU/mL); No antiviral treatment or drug discontinuation for more than 6 months before enrollment; Willing to cooperate to complete the research process and sign informed consent form; No drugs affecting the intestinal flora were used within 2 weeks before enrollment.

#### 2.2.2 Exclusion criteria

Pregnant or lactating women; combined with other hepatophilic virus infections (confirmed by detection of corresponding viral markers); suffering from mental disease or cognitive disorder and unable to cooperate; history of alcohol abuse (≥40 g pure alcohol per day for males and ≥20 g pure alcohol for females for more than 5 years) or drug abuse; has received a liver transplant; is allergic to or intolerant of test methods for intestinal flora (e.g., fecal sample collection process, high-throughput sequencing reagents, etc.).

### 2.3 Clinical feature detection method

#### 2.3.1 Degree of appetite loss

The degree of anorexia was assessed using the Visual Analogue Scale (VAS). A 10 cm straight line was drawn on the paper, with both ends marked as “normal appetite (0 point)” and “no appetite at all (10 points)” respectively, for patients to mark a point on the straight line according to their appetite, so as to quantify the degree of anorexia. A score greater than 5 indicated a marked decrease in appetite.

#### 2.3.2 Degree of fatigue

The Fatigue Severity Scale (FSS) was used to quantify the symptoms of fatigue. The scale consisted of nine items, with each item graded from 1 (strongly disagree) to 7 (strongly agree), with a total score of 9–63.

#### 2.3.3 Jaundice degree

The degree of jaundice was accurately assessed by measuring serum total bilirubin (TBIL) and direct bilirubin (DBIL).

### 2.4 Laboratory test methods

All biological samples were processed following standardized clinical laboratory protocols. Venous blood samples were collected in appropriate anticoagulant tubes (EDTA-K2 for virologic tests, sodium citrate for coagulation tests) and centrifuged (3,000 rpm, 10–15 min) to separate plasma/serum, which was either analyzed immediately or stored at recommended temperatures (2–8°C for ≤24 h; −20°C for long-term storage).

Liver function tests (ALT, AST, TBIL, and DBIL) and albumin/globulin measurements employed automated biochemical analyzers using manufacturer-certified reagents. HBV DNA quantification used Roche Cobas TaqMan HBV Test (lower limit 20 IU/mL) with qPCR. Serological markers (HBsAg, HBeAg) were detected by ELISA. PT/INR measurements utilized fully automated coagulation analyzers.

### 2.5 Intestinal flora distribution detection method

Fecal samples were collected from patients under fasting conditions using sterile containers, with approximately 2–3 g of fecal material obtained from the central portion to avoid contamination. Samples were immediately stored at −80°C until processing. DNA extraction was performed using the QIAamp DNA Stool Mini Kit (Qiagen) following manufacturer’s protocols. DNA concentration and purity were verified using a Nanodrop spectrophotometer (A260/A280 ratio 1.8–2.0; concentration 20–50 ng/μL).

The V3–V4 region of the 16S rRNA gene was amplified using 338F-806R primers under standard PCR conditions (95°C for 5 min; 30 cycles of 95°C/30 s, 55°C/30 s, 72°C/30 s; final extension at 72°C for 10 min). Purified amplicons were sequenced on the Illumina MiSeq platform (2 × 300 bp) with 50,000–100,000 reads per sample. Raw data were quality-filtered (Q30) using FastQC, and OTUs were clustered at 97% similarity with UPARSE. Taxonomic annotation was performed against the Silva database. Alpha-diversity indices (Shannon-Wiener, Simpson, Chao1, and ACE) and relative abundances at phylum/genus levels were calculated.

### 2.6 Antiviral treatment

Antiviral therapy with nucleoside (acid) analogs, including entecavir (ETV) and tenofovir disoproxil fumarate (TDF), has been used in patients with chronic hepatitis B in this study. For treatment-naive patients, entecavir was given at a dose of 0.5 mg/ day and tenofovir disoproxil fumarate at 300 mg/day. During the treatment, the patients were required to take quantitative drugs strictly according to the prescribed timing to ensure the effectiveness and stability of the drugs. The treatment cycle should be at least 48 weeks. The adverse drug reactions of the patients should be closely observed during the treatment. For example, entecavir may cause adverse reactions such as headache, fatigue and vertigo, and tenofovir disoproxil fumarate may cause renal impairment and hypophosphatemia. Medical staff regularly carry out relevant examinations on patients, such as renal function tests, in order to timely detect and deal with possible adverse drug reactions.

### 2.7 Treatment effect evaluation method

Treatment effectiveness was evaluated through a composite endpoint incorporating both clinical and objective measures: (1) symptom improvement (resolution of fatigue, jaundice, and liver discomfort), (2) virological response (≥2 log10 IU/mL decrease in HBV DNA or undetectable levels), (3) biochemical response (ALT normalization ≤40 U/L), and (4) serological response (HBeAg loss or seroconversion where applicable). Patients meeting ≥3 criteria were classified as having effective treatment.

Effective: (1) HBV DNA reduction ≥2 log10 IU/mL or undetectable levels (<20 IU/mL); (2) ALT normalization (<40 U/L for males, <35 U/L for females); (3) Improvement in clinical symptoms (e.g., fatigue, jaundice).

Invalid: (1) HBV DNA reduction <2 log10 IU/mL; (2) Persistent ALT elevation; (3) Worsening symptoms or new complications (e.g., ascites).

### 2.8 Statistical methods

SPSS 26.0 software was used for statistical analysis. Categorical data were analyzed using the chi-square test, and continuous data were analyzed using the Student’s *t*-test or Mann–Whitney *U* test, as appropriate. For multivariate analysis, logistic regression was employed to identify independent risk factors for antiviral treatment failure. Variables with a significance level of *p* < 0.05 in univariate analysis were included in the multivariate logistic regression model. The nomogram prediction model was constructed based on the results of multivariate logistic regression using the “rms” package in R software (version 4.2.1). The predictive performance of the nomogram was evaluated using the area under the receiver operating characteristic curve (AUC-ROC), calibration curves, and the Hosmer–Lemeshow test. Decision curve analysis (DCA) was performed to assess the clinical utility of the model. A two-tailed *p* < 0.05 was considered statistically significant.

## 3 Results

### 3.1 Comparison of antiviral treatment effects, clinical characteristics and intestinal flora distribution parameters between the training set and the verification set

Forty-five patients (25.71%) in the training set were ineffective, and 19 patients (25.33%) in the verification set were ineffective. The training (*n* = 175) and verification (*n* = 75) sets showed no significant differences in demographic (age, gender, BMI), clinical (liver function tests, viral load), or microbiota parameters (all *p* > 0.05), confirming their comparability. Notably, key predictors like AST (42.14 ± 15.10 vs. 45.64 ± 16.34 U/L, *p* = 0.103) and Shannon-Wiener index (3.47 ± 0.60 vs. 3.61 ± 0.87, *p* = 0.181) were well-balanced, supporting the validity of subsequent model development and validation, as shown in Table 1.

**Table 1:** Comparison of clinical characteristics and intestinal flora distribution parameters between the training set and the verification set.

Project		Training set (*n* = 175)	Verification set (*n* = 75)	Statistical values	*p*-value
Age (years)		46.09 ± 10.11	45.57 ± 10.03	0.368	0.713
Gender	Male	104	40	0.799	0.371
	Female	71	35		
BMI (kg/m^2^)		23.44 ± 3.78	24.01 ± 3.67	1.085	0.279
Course of disease (years)		7.47 ± 4.15	7.34 ± 5.02	0.196	0.845
Degree of appetite loss		5.82 ± 4.93	6.01 ± 4.53	0.287	0.774
Degree of fatigue		37.20 ± 6.39	36.34 ± 6.57	0.975	0.332
TBIL (μmol/L)		16.96 ± 7.04	16.43 ± 6.64	0.557	0.578
DBIL (μmol/L)		6.38 ± 3.13	6.66 ± 3.56	0.601	0.548
ALT (U/L)		48.22 ± 20.55	47.64 ± 18.34	0.211	0.833
AST (U/L)		42.14 ± 15.10	45.64 ± 16.34	1.638	0.103
ALB (g/L)		41.77 ± 4.19	42.49 ± 4.17	1.251	0.212
GLB (g/L)		28.78 ± 5.25	29.06 ± 5.03	0.395	0.693
Hepatitis B virus DNA quantification (IU/mL × 10)		0.18 ± 0.10	0.16 ± 0.08	0.838	0.403
HBsAg (S/CO)		12.74 ± 4.77	11.64 ± 5.12	1.629	0.105
HBeAg (S/CO)		8.55 ± 3.54	7.69 ± 4.65	1.433	0.155
PT (seconds)		12.57 ± 2.83	11.84 ± 3.64	1.717	0.087
INR		1.13 ± 0.17	1.16 ± 0.19	1.535	0.126
Shannon-Wiener index		3.47 ± 0.60	3.61 ± 0.87	1.345	0.181
Simpson index		0.37 ± 0.12	0.41 ± 0.24	1.666	0.099
Chao1 index		148.07 ± 29.64	155.24 ± 30.21	1.742	0.083
ACE index		157.09 ± 34.92	152.24 ± 33.61	1.017	0.310
Relative abundance of *Chlamydoma*		36.50 ± 9.01	37.65 ± 10.21	0.888	0.375
Relative abundance of *Bacteroides*		39.30 ± 10.16	40.54 ± 10.32	0.878	0.381
PCA clustering separation	Clear	90	42	0.440	0.507
	Not obvious	85	33		

Further analyses were performed to compare additional baseline characteristics, such as comorbidities (e.g., diabetes, hypertension) and medication history (e.g., prior use of probiotics or antibiotics), between the training and validation sets. No significant differences were observed (all *p* > 0.05), confirming the comparability of the two groups and supporting the validity of the model.

### 3.2 Comparison of clinical characteristics and distribution parameters of intestinal flora between the ineffective and effective groups in the training set

In the training set, the results of single factor analysis showed that the treatment ineffective group and the effective group had statistically significant differences in ALT, AST, hepatitis B virus DNA quantification, Shannon-Wiener index, Simpson index, Chao1 index, ACE index, relative abundance of Firmicutes, relative abundance of Bacteroides and PCA clustering separation (*p* < 0.05), as shown in Table 2.

**Table 2:** Comparison of clinical characteristics and distribution parameters of intestinal flora between the ineffective and effective groups.

Project		Ineffective group (*n* = 45)	Effective group (*n* = 130)	Statistical values	*p*-value
Age (years)		48.49 ± 12.35	45.25 ± 9.12	1.862	0.064
Gender	Male	28	76	0.196	0.658
	Female	17	54		
BMI (kg/m^2^)		24.36 ± 4.25	23.12 ± 3.56	1.910	0.058
Course of disease (years)		8.45 ± 5.67	7.13 ± 3.45	1.472	0.147
Degree of appetite loss		7.03 ± 5.56	5.40 ± 4.65	1.926	0.056
Degree of fatigue		38.80 ± 6.24	36.64 ± 6.37	1.969	0.051
TBIL (μmol/L)		18.72 ± 10.56	16.34 ± 5.23	1.452	0.152
DBIL (μmol/L)		7.14 ± 4.67	6.12 ± 2.34	1.393	0.170
ALT (U/L)		55.62 ± 20.44	45.67 ± 20.34	2.857	0.005
AST (U/L)		47.34 ± 13.54	40.34 ± 15.23	2.730	0.007
ALB (g/L)		40.75 ± 5.34	42.13 ± 3.67	1.597	0.116
GLB (g/L)		30.05 ± 6.78	28.35 ± 4.56	1.565	0.123
Hepatitis B virus DNA quantification (IU/mL × 10)		0.23 ± 0.12	0.15 ± 0.08	4.308	0.001
HBsAg (S/CO)		13.92 ± 5.24	12.34 ± 4.56	1.928	0.055
HBeAg (S/CO)		9.45 ± 3.65	8.25 ± 3.45	1.925	0.058
PT (seconds)		13.27 ± 3.45	12.34 ± 2.56	1.660	0.102
INR		1.17 ± 0.23	1.12 ± 0.15	1.395	0.168
Shannon-Wiener index		3.22 ± 0.67	3.55 ± 0.56	3.225	0.002
Simpson index		0.43 ± 0.13	0.35 ± 0.12	3.832	0.001
Chao1 index		140.56 ± 25.34	150.67 ± 30.65	1.987	0.048
ACE index		146.45 ± 30.56	160.78 ± 35.67	2.405	0.017
Relative abundance of *Chlamydoma*		39.56 ± 10.23	35.45 ± 8.34	2.690	0.008
Relative abundance of *Bacteroides*		35.34 ± 8.56	40.67 ± 10.34	3.101	0.002
PCA clustering separation	Clear	30	60	5.631	0.018
	Not obvious	15	70		

### 3.3 Analysis of risk factors for the effect of antiviral treatment

Treatment effect was taken as the dependent variable (0 = effective, 1 = ineffective), and the factor with *p* < 0.05 in single factor analysis was taken as the covariate. Further multivariate logistic regression analysis showed that AST, hepatitis B virus DNA quantification, Shannon-Wiener index, Simpson index, the relative abundance of Firmicutes and Bacteroides were the independent risk factors for the ineffectiveness of antiviral therapy (*p* < 0.05), as shown in Table 3.

**Table 3:** Logistic regression analysis of risk factors for the effect of antiviral therapy.

Project	*B*	S.E.	Wald	*p*	OR	95% CI
ALT	0.024	0.012	3.782	0.052	1.024	1.000–1.050
AST	0.051	0.018	8.036	0.005	1.052	1.016–1.090
Hepatitis B virus DNA quantification	10.103	2.542	15.799	0.001	24414.630	167.5–35,578
Shannon-Wiener index	−1.103	0.404	7.461	0.006	0.332	0.150–0.732
Simpson index	4.126	1.984	4.325	0.038	61.913	1.268–3022.869
Chao1 index	−0.011	0.009	1.424	0.233	0.989	0.971–1.007
ACE index	−0.013	0.007	3.192	0.074	0.987	0.973–1.001
Relative abundance of *Chlamydoma*	0.056	0.028	3.895	0.048	1.058	1.000–1.118
Relative abundance of *Bacteroides*	−0.058	0.024	5.895	0.015	0.944	0.901–0.989
PCA clustering separation	1.018	0.525	3.761	0.052	2.767	0.989–7.738
Constant	−1.196	2.566	0.217	0.641	0.302	

### 3.4 Establishment of nomogram prediction model for antiviral treatment effect

**Figure 1:**
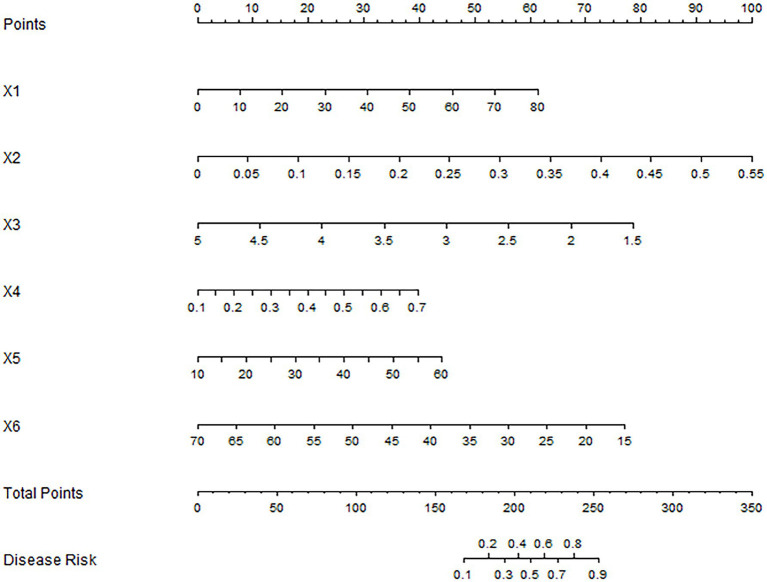
Nomogram of antiviral treatment effect prediction model. X1–X6 were: AST, hepatitis B virus DNA quantification, Shannon-Wiener index, Simpson index, relative abundance of Sclerotinia wall and relative abundance of Bacteroides immitis, respectively.

Based on the independent risk factors identified by multivariate logistic regression analysis, a nomogram prediction model for the effect of antiviral treatment was constructed. Each independent risk factor in the model was scored, and the total score for predicting the effect of antiviral treatment was calculated, which was reflected in the prediction of the incidence of ineffective antiviral treatment. The higher the total score was, the higher the accuracy was in predicting the effect of antiviral treatment, as shown in Figure 1.

### 3.5 Evaluation and validation of predictive models for the effect of antiviral therapy

**Figure 2:**
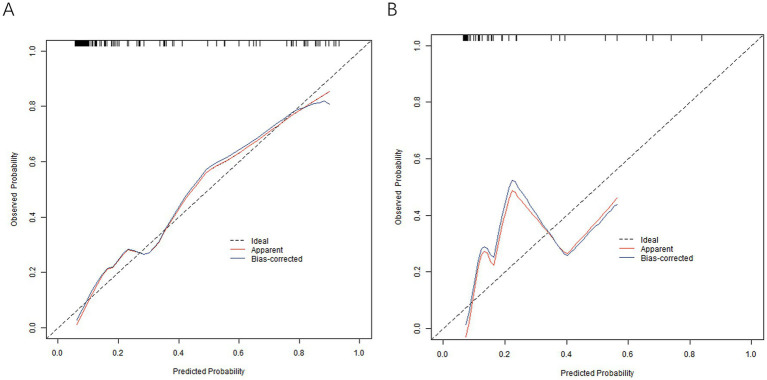
Calibration curve in the training set **(A)** and the verification set **(B)**.

**Figure 3:**
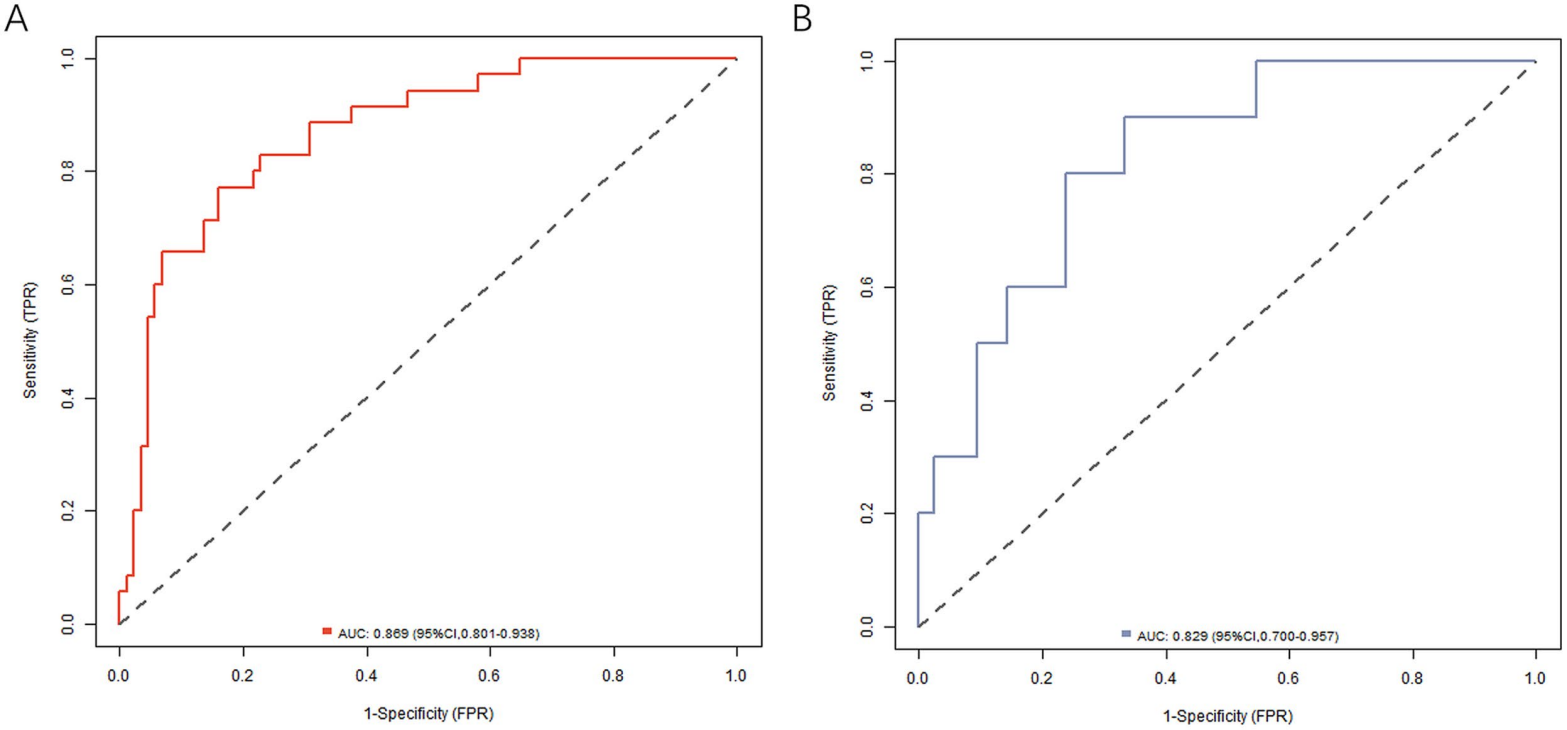
ROC curve in the training set **(A)** and the verification set **(B)**.

In the training and validation sets, the nomogram model *C*-index was 0.866 and 0.816, respectively, the calibration curve showed mean absolute errors of 0.125 and 0.122, respectively, for the predicted and actual values, and the Hosmer–Lemeshow test was *χ*^2^ = 5.937, *p* = 0.654 and *χ*^2^ = 4.886, *p* = 0.770, respectively. The ROC curves were displayed in the training and validation sets, and the AUC of the nomogram model for predicting the effect of antiviral therapy was 0.869 (95% CI: 0.801–0.938) and 0.829 (95% CI: 0.700–0.957), respectively, with sensitivity and specificity of 0.771, 0.841, and 0.600 and 0.786, respectively. The calibration curves are shown in Figure 2 and the ROC curves are shown in Figure 3. While the nomogram demonstrated good discrimination (AUC >0.8) in both training and validation sets, the validation cohort was derived from the same single-center population, which may limit generalizability. Further multi-center studies with larger cohorts are needed to confirm the model’s robustness across diverse clinical settings.

### 3.6 Analysis of decision curve of nomogram prediction model for antiviral treatment effect

**Figure 4:**
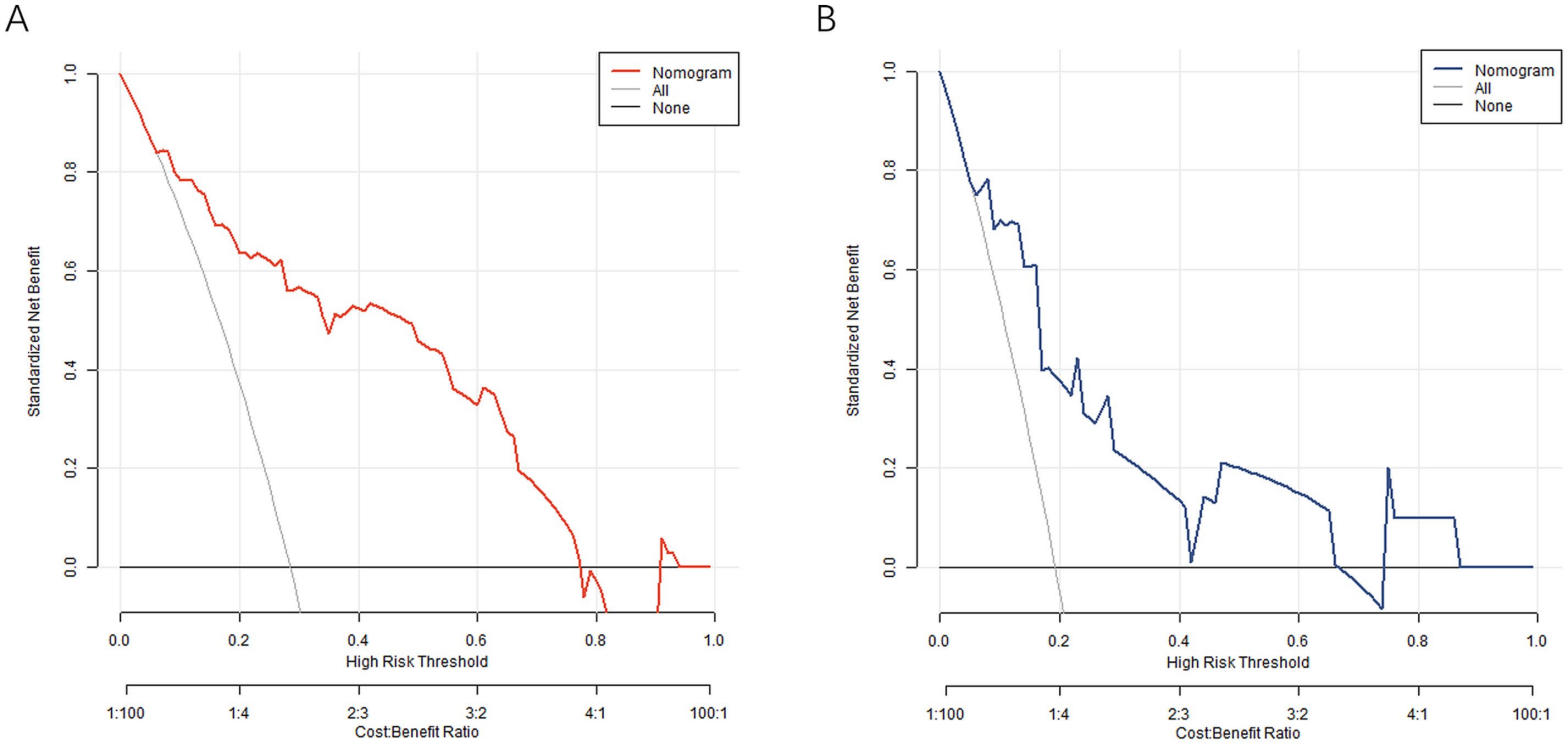
Decision curve in the training set **(A)** and the verification set **(B)**.

The decision curve showed that when the threshold probability was within the range of about 0.05–0.95, the nomogram model constructed in this study would have more clinical benefits for predicting the prognosis of chemotherapy, as shown in Figure 4.

## 4 Discussion

Chronic hepatitis B treatment remains a major focus in clinical research. Although antiviral therapy is the key measure, the significant difference in the treatment effect has prompted researchers to explore more accurate prediction indicators and methods (14). In recent years, the correlation between intestinal flora and chronic hepatitis B has been gradually deepened, opening a new perspective for the diagnosis and treatment of diseases. In this study, we successfully constructed a nomogram prediction model based on the clinical characteristics of patients with chronic hepatitis B and the distribution of intestinal flora. The model showed good prediction performance in both the training set and the verification set, and it had many important meanings and values.

From the perspective of clinical characteristics, we found that such indicators as AST and hepatitis B virus DNA quantification were independent risk factors for the failure of antiviral treatment. AST is a sensitive indicator of hepatocyte injury, and its elevated level reflects severe hepatic inflammatory activity, which may affect the effect of antiviral therapy (15). Hepatitis B virus DNA quantification directly reflects the virus replication activity, and high viral load often means that the virus is difficult to be effectively inhibited, which is closely related to the ineffective treatment (16). These findings align with prior evidence, underscoring the critical role of closely monitoring liver function indicators and viral load in the treatment process of chronic hepatitis B, and providing a key basis for the adjustment of clinical treatment options. For example, patients with persistently high levels of hepatitis B virus DNA quantification and abnormally high AST may need to consider replacing more effective antiviral drugs or other treatment modalities in combination to increase the success rate of treatment. The distribution of intestinal flora also plays an important role in the prediction of the efficacy of antiviral treatment for chronic hepatitis B. The Shannon-Wiener index and Simpson index determined in this study reflected the diversity of intestinal flora, while the Chao1 index and ACE index reflected the richness, as well as the relative abundance of Firmicutes and Bacteroides were related to the treatment effect. Although our study identified associations between intestinal flora (e.g., Firmicutes/Bacteroides ratio) and treatment response, the observational design precludes causal inferences. Mechanistic studies are warranted to explore whether microbiota alterations directly influence antiviral efficacy or merely reflect disease severity. Patients with chronic hepatitis B have an imbalanced intestinal flora, with decreased diversity and abundance, and an imbalanced ratio of beneficial to harmful bacteria. For example, the increase in the relative abundance of Firmicutes and the decrease in the relative abundance of Bacteroides may interfere with the normal process of antiviral therapy by affecting the intestinal barrier function, immune regulation, and interaction with hepatitis B virus (17). The intestinal barrier is damaged due to the imbalance of intestinal flora, and the bacterial translocation causes the aggravation of liver inflammatory reaction, leaving the liver in a state of continuous injury, which is not conducive to the effect of antiviral therapy. At the same time, abnormal changes in the metabolites of the intestinal flora can regulate the immune microenvironment of the liver and inhibit the effective antiviral immune response of the body (18). This suggests that besides the treatment for the virus itself, the regulation of intestinal flora may become a new adjuvant treatment strategy in the treatment of chronic hepatitis B. In the future, the feasibility of improving the intestinal flora and thus the antiviral treatment effect by means of probiotics, prebiotics or fecal bacteria transplantation will be further explored.

The nomogram prediction model developed in this study integrated clinical characteristics and multidimensional intestinal flora data, demonstrating significant advantages over single-index predictions. Its visual characteristics enable clinicians to more intuitively and quickly accurately predict the effect of antiviral treatment according to the indicators of patients, which is conducive to the development of personalized treatment. For example, for patients with poor therapeutic prediction, tighter monitoring plans can be planned in advance, therapeutic drugs can be adjusted, or other treatment measures can be combined, so as to avoid unnecessary treatment delay and resource waste. For patients with good predictive therapeutic effects, the follow-up procedure can be appropriately simplified to reduce the economic and psychological burden on patients. To address potential concerns regarding the representativeness of our sample, we expanded our baseline comparisons beyond the initially reported parameters. The inclusion of additional variables, such as lifestyle factors (e.g., smoking, alcohol consumption) and detailed medication histories, further validated the similarity between the training and validation sets. This comprehensive approach strengthens the reliability of our nomogram model and its applicability to diverse clinical settings. While this study provides valuable insights, several limitations should be acknowledged. First, as a single-center study, the generalizability of our findings may be limited. Although we rigorously controlled for confounding factors and ensured internal validity through randomized training/validation sets, external validation in multi-center cohorts with diverse populations is necessary to confirm the model’s broader applicability. Second, while our references cover key aspects of CHB and gut microbiota, future studies could benefit from citing more high-impact journals to strengthen theoretical foundations. Lastly, although we employed standardized protocols for microbiota analysis, functional metagenomics or metabolomic profiling could provide deeper mechanistic insights in future research. Third, while we incorporated both clinical and microbial variables, the predictive performance might be improved by including serial measurements of HBV DNA and liver enzymes during treatment. Fourth, our symptom-based effectiveness assessment could be strengthened by adding quantitative measures like the Chronic Liver Disease Questionnaire (CLDQ) to standardize symptom reporting. Finally, the observational nature of our microbiota data cannot establish whether microbiome alterations are drivers or consequences of treatment outcomes. Second, although the commonly used 16S rRNA gene sequencing technology is adopted for the detection of intestinal flora, it may not fully and accurately reflect the functional status of intestinal flora and its complex interaction with the host (19). Future studies may consider the combination of metagenomics, metabolomics and other multi-omics technologies to further explore the mechanism of intestinal flora in the antiviral treatment of chronic hepatitis B and further optimize the prediction model. While our study identified significant associations between specific microbiota features (e.g., Shannon-Wiener index, Firmicutes/Bacteroides ratio) and treatment outcomes, we acknowledge that the causal relationships between gut microbiome composition and antiviral response remain to be fully elucidated (20). These findings should be interpreted as preliminary evidence requiring validation through mechanistic studies. Future research combining metagenomic sequencing with metabolomic profiling may help clarify whether these microbial signatures play active roles in modulating treatment response or simply reflect underlying host-pathogen interactions. In addition, in this study, the antiviral effect of nucleoside (acid) analogs was only predicted, and the applicability of other antiviral drugs or combination therapy was not clear, which needs further research and development.

In summary, the nomogram prediction model constructed in this study provides a new and effective tool for predicting the effect of antiviral treatment for chronic hepatitis B and powerful support for clinical personalized treatment. However, it still needs further in-depth study and improvement in many aspects to better serve the clinical treatment and management of patients with chronic hepatitis B and promote the development of precision medical treatment of chronic hepatitis B.

## Data Availability

The raw data supporting the conclusions of this article will be made available by the authors, without undue reservation.
